# The Nursing Supplement

**Published:** 1889-11-09

**Authors:** 


*The Hospital,*
November 9, 1889.

*Extra Supplement*

### The Nursing Mirror.

All Contributions for this Supplement should be addressed to the Editor, The Hospital, 139-140, Salisbury Court, Fleet Street, London, E.C., and should have the word "Nursing" plainly written in left-hand top corner of the envelope.

### En Passant.

A TRAVELLED STORY.—After a preliminary visit to America, a tour in Australasia, and a frequent appearance in China, the story of "'Opkins," which appeared in The Hospital for September, 1888, has found a home on the programme of Mr. and Mrs. Kendal's performances in the States. Imagine the horror of one of our staff, who had been haunted by "'Opkins" for a year, and who was seeking a total change in Fifth-avenue Theatre, to find the ubiquitous story staring him in the face when he opened his programme!

SHORT ITEMS.—No sisters will in future be received into the Army Medical Service who have not had three years' training in a good general hospital.—The Sisters of Providence, Bartrams, Hampstead, are a Catholic religious order who include nursing amongst their good works.—The death is announced of Nurse Gale, formerly of Pendlebury and The London.—The Little Sisters of the Poor have lately opened two more homes in the United States, one at Alleghany for 250 inmates, and one at Germantown.—Mr. Mansell Moullin will deliver the lectures on surgical nursing at the London Hospital this session, instead of Mr. Treves.—Miss Barnett is delivering a series of lectures on nursing at Richmond, Surrey.—Miss Whistler, sister of the artist, is a naval nursing sister at Chatham.

A LECTURE TO NURSES.—Last Friday evening a lecture on "Early Symptoms of Mental Disorder after Confinement, and their Treatment," was delivered by Dr. Savage (late physician and superintendent of Bethlem Royal Hospital), at the Midwives' Institute and Trained Nurses' Club, 15, Buckingham-street, Strand. The room was crowded; the audience consisting entirely of midwives and nurses. The lecturer described the various kinds of insanity that may attack the puerperal woman, with their pre-disposing and exciting causes, their symptoms and treatment, and gave much valuable instruction to nurses on the management of these difficult cases. Dr. Savage said that the average duration of puerperal mania was from three to six months. Out of 100 cases five die, 20 remain incurable, and 75 recover. One interesting fact mentioned was that puerperal mania occurs more often after normal than after instrumental labour.

NURSING SISTERS AT PLAISTOW.—Hospitals are all far away from Plaistow, and the people beyond the reach of the East-end charities and district nurses. Early in this year two nurses started work in Plaistow, E., having for their headquarters St. Mary's Mission-house. A small crèche was opened, and as much parish nursing and midwifery undertaken as possible, first with the help of one more nurse, and now with a still increasing staff of nine workers. Some are training in midwifery work, others much younger are training in the day nursery, finding there a useful sphere while waiting to enter hospitals. The crèche has been enlarged and a new house opened for a nurses' home. Infants are admitted from two weeks old. There is plenty to do and plenty to learn, and of work there is no limit. The pretty crèche and its still prettier little inmates, with their red overalls, or red jerseys and tam o' shanters, are well worth a visit, and Plaistow, over the borders, is only fifteen minutes from Fenchurch-street Station. Visitors are gladly welcomed any afternoon. Helpers are still needed, especially young lady nurses for the children's work.

AN ABERDEEN MATRON.—The opening address in clinical surgery delivered at Aberdeen on October 25 by Dr. Garden, dealt first with Prof. Struthers and his work, and secondly with the progress of Aberdeen Royal Infirmary. In telling the history of this last Dr. Garden said: "Under the superintendence of Miss Lumsden, who, at much personal sacrifice—for she had well earned leisure after her great labour in founding and establishing the Sick Children's Hospital—put her services, and that gratuitously, too, at the disposal of the managers, the internal economy has become most efficient, while an entirely new nursing system has been introduced. We have now what, I think, is the best possible system of nnrsing. Hitherto Miss Lumsden and her staff have been working, and still work, at great disadvantage on account of insufficient accommodation. Without ample accommodation for the administrative staff no hospital can be efficiently or economically managed." This was, indeed, well-earned public praise.

THE PENSION FUND.—Amongst the metropolitan institutions which have become affiliated to the National Pension Fund for Nurses are Guy's Hospital, the London Hospital, St. Mary's Hospital, the Seamen's Hospital, Greenwich, the Mildmay Nursing Institution, and the Nursing Sisters of St. John the Divine. In the provinces, the Royal Hants County Hospital, Winchester, the Warneford Hospital, Leamington, and the Bristol Nurses' Training Institution have also become affiliated to the fund. The East London Nursing Society, the Liverpool Pension Fund, the Bristol Royal Infirmary, and others have the matter at present under consideration, but the details have not yet been settled. Among other donors to the fund we may mention Mr. John Norbury, Mr. A. Mocatta, Mr. M. Van Raalte, the Countess of Rosebery, Miss Spigg, and the executors of the late Captain A. C. Rae. We wonder how those foolish persons feel who twelve months ago announced to nurses that the fund would never be established? They were so sure registration would be established by now, and the Pension Fund a failure, that in future it would seem safe always to believe the opposite to what they say.

THE ASYLUMS BOARD ON MATRONS.—At the fortnightly meeting of the Metropolitan Asylum's Board last Saturday, the chairman, Sir E. Galsworthy, said he had received from Dr. J. H. Bridges, medical inspector to the Local Government Board, suggestions as to the qualifications which should be looked for in the candidates for the office of matron in the fever hospitals. He proposed that the communication should go to the General Purposes Committee to communicate with the Hospital Committees. Dr. Bridges said the question was specially important at the present time, when the managers were considering the expediency of admitting students of medicine to their hospitals. The nursing staff, in such a case, should be under a system of government and discipline at least as good as that which obtained in the general hospitals of London. The nurses should be under the superintendence of a highly qualified officer of their own sex. Subordination to a matron was only conceded by a nursing staff where the matron herself possessed the skill and experience appertaining to nursing. His own experience of the twenty-four poor-law infirmaries of the metropolis endorsed this. It had worked with admirable effect at the St. Marylebone Infirmary, which was a model of good management in all that referred to the nursing staff. This is news which will be welcome to all true nurses.

### Three Finches.

"I," SAID Mollie Finch, pompously, "I have made up my mind what I shall be when I'm grown up."

"What?" asked her two sisters, eagerly.

The three small maidens, each armed with a beloved and much battered old doll, sat in their special corner of mother's room—the wide bay-window recess. Three little Finches were they, human birds in the home-nest.

"A washerwoman!" announced Mollie, slowly, and with as much importance as if she had said an empress or a Patti. Then the sudden tears of vexation sprang into her clear, round eyes at the burst of derision her exalted idea of a career elicited. "Why not?" she went on with warmth. "It's a most beautiful life, a washerwoman's. Oh, I should love to be one! My washing-days are the happiest of all the week." And she examined the frills and skirts of her waxen baby anxiously, hoping to catch sight of a speck which might excuse an extra field-day of her favourite diversion.

"Well, then," said Enid, the second little Finch, "you will be a misgrace to the family, that's all. And I shan't never come to see you, for when I grow up I shall marry a duke, and have a carriage-and-four, and always have lump sugar in my tea instead of that nasty brown stuff we have in the nursery." And Miss Enid Finch stretched out her inches to their utmost.

"I don't care," said Mollie, stoutly; "driving isn't half such fun as wringing and hanging out clothes. Oh, that's just lovely!" she wound up, ecstatically.

"What shall you be, Goldy?" asked Enid, turning scornfully from the future black sheep of the family to her other little sister.

His Goldfinch, as her father called her, was dreamily looking out of her blue eyes into the misty, hazy middle-distance and the faint line of hills beyond—the view from mother's window. The first thing you were forced to notice about Goldy was her shining locks rippling down over the slight, little form—a priceless mantle of hair that glittered in the sunlight and won for her the home name. "She's our flower," the father and mother would tell each other with secret pride. "There will be no one like her in all the county when she grows up to be a woman"; but they wished Goldy was not so still, so silent—wished that she would run about more, like her sisters did, even that she would join in their sparring matches, their flying quarrels. She is too good, was the thought unspoken, and full of fear deep down in her mother's heart, perhaps, it may be that she is only lent to us in order to lead us up higher. Oh, if she is to be taken, she would add passionately, God grant it may not be until I am yonder, ready to meet and greet her!

"What shall I be?" said Goldy, coming back from who can tell where a little child's spirit wanders into the warm, comfortable room. "Oh, dear, how do I know!" and she gave her baby a squeeze. Goldy's doll was quite the most battered of the three, owing to the wealth of affection lavished upon it. "I think," said the puzzled, little maid, at last, "I'd like to be a bird—to be something with wings, you know."

"Don't be silly!" put in Mollie, always practical; "we're talking real—not pretending. Say, what should you like to be when you're a grown-up?"

"Well," mused Goldy, quite cornered, "I expect I'd like to be a help. Mother and nursie were talking about helps when they put me to bed the last time I had a cold"—poor Goldy's colds were periodical events—"and mother said they were the best kind of women ever invented, but nursie didn't think so at all. You see, Mollie, I could come and help you in your house, for washerwomen are always very poor, and I could go to help Enid, if she grows up a duchess, in some way; and I could help mother to housekeep. Yes, that's what I shall be—a help," ended Goldy with gentle decision.

"I don't think that's the kind of help mother and nursie were talking about," said Mollie doubtfully. "But, Goldy, I do think your plan is beautiful; and now I must wash my Geraldine-Alice's muslin blouse." And forthwith the little parliament dissolved.

Quicker, faster speed the years. There is no dallying, no loitering with old Time, as it hurries by, uninterrupted by sorrows and joys, pressing forward the young, carrying along the old—where?

The home-nest of the three little Finches is empty. Time's work, that. The birds have flown out to try their wings, and, after the manner of birds, have not returned. Nature's way, of course, to which even the deserted parents become reconciled.

Mollie Finch has achieved what is called a success in life. Her childish aspirations after the useful have melted into thin air. Mollie has made a grand marriage, and is a very great lady, indeed—a society queen—her life is filled up with "pomps and vanities." The energy natural to her finds its outlets in a round of ceaseless gaities, instead of wringing and hanging out clothes—that El Dorado of a life, pictured and coveted in her happy childhood. Jewels, show, emulation, triumphs, supply the daily food of her heart and mind. Almost, that is, not quite, for there are hours when a "still small" voice steals into her ears, and gently draws her thoughts from the bewildering, hurrying world.

The memory of a fair, meek sister—the flower of them all—comes, at times, to Mollie, and there is an abrupt pull-up in her headlong career while she broods over the past with its simple joys. She will turn over a new leaf, she tells herself. She will live a purer, higher life. She will "take heed unto the thing that is right," and have dealings only with such ways as are "lovely and of good report," and Mollie rises up to perform some great, good work, such as her wealth enables her to carry out. Thus is Goldy fulfilling the wish of her heart—she is a help to Mollie.

And Enid's dream. Has it come to pass? Scarcely. In one of London's great hospitals moves about a tall, stately young nurse, one of the brightest, the most intelligent, that has ever entered the profession. Enid Finch grew up, and left far behind her the childish desire for a high position in life. A loftier ambition seized her—Enid must, of necessity, aspire, it being her nature—she would go out into the world of suffering and heal the sick. Such a nature and the physical power to fulfil it are not given to all women, not even to many, but Enid was one of the few, and she wisely gave herself to the destiny for which she was most fitted.

Occasionally, it is true, there are fitful longings for "lump sugar instead of the nasty brown stuff we get in the nursery," but Goldy's help steps in here also. Remembrance of the beautiful, gentle spirit, ever ready to sacrifice itself for others while it had the strength, and, when that was gone, so willing to give up life in its springtime because her Father in heaven deemed it best, stirs the better nature of Nurse Enid, and she throws herself with a new-born vigour into some urgent case.

"I shall press to the front rank of the workers," she murmurs, with set lips; "my meaner self must not drag me back while 'the maimed, the halt, and the blind' are crying out for my help."

And what of Goldy herself? For human eyes there is but a white cross by a grassy mound. Her work of helping goes on; her influence lives still in the happy lives of her sisters; but her yearning to be something with wings is granted, but Goldy herself is "absent in the body."

### Examination Questions.

TWENTY answers were received to the examination question asked in THE HOSPITAL of 12th Oct., and, after considerable difficulty, we have decided to award the prize to Nurse Rose, Union Infirmary, Cardiff, to whom we have sent a copy of Murrell's "Masso-Therapeutics." Several of the answers received were almost as good as those of Nurse Rose; indeed, we wished we could have selected the best bits from each paper to make one perfect answer. A nurse who wrote clearly on one point, was so often careless about another point. Miss Hotham sent an excellent paper, which was, however, too like a treatise on the comparative merits of different antiseptics. Perhaps the question given was rather vague, but it was a genuine one, set by a well-known surgeon to the nurses of a large training school. The question for November is: "Describe the composition of bone; name the four classes of bone, and give an example of each." Answers must reach our office by November 23; they must be short and clear, written on one side of the paper only, and accompanied by the writer's full name and address. The October prize answer will be published next week.

### The Management of Children.

### I.—THE FEEDING OF INFANTS.

We have upon our table two books of interest to nurses and mothers, and to women generally. The first is "The Management of Children," by "A Mother," published by Messrs. Churchill. The second is "A Handbook for the Nursing of Sick Children," by Miss C. J. Wood, published by Messrs. Cassell and Co. The first is a substantial and readable volume full of information and of practical hints; the second is a technical work on nursing, excellent in its way, yet missing many points made plain in the larger book. For instance, with regard to the feeding of infants, Miss Wood gives a few pages, in the course of which she tells the ordinary amount of milk given to a child of a given age, mentions the best chemical foods in a footnote, and gives her verdict with regard to the shape of the bottle. But "A Mother" devotes some 200 pages to the feeding of infants, and gives an analysis of most of the foods she mentions. Also she gives in footnotes her authority for every statement. That children should be fed regularly is a fact which needs impressing more on parents than on trained nurses, but many a private nurse might be glad of the knowledge about the preparation of the food which A Mother gives, and the manner of giving it. How life-like is the picture of the poor baby, who, instead of being allowed to have its bottle in peace, is sat bolt upright at intervals, and thumped on the back to "break up the wind!" This is a performance we have seen gone through by nurses from celebrated London institutions, supposed to have been highly trained in monthly nursing. The three essentials of rearing by hand are given as: (1) Exactitude in preparing the food so that it is always the same, and always the same heat; (2) cleanliness as regards the vessels the food is prepared and given in; (3) regularity in giving food, and giving it sufficiently sustaining, and in sufficient quantity. That no nursery should be without Dr. Forbes's thermometer for testing the heat of the food, is also a good rule insisted on. With regard to the cleaning of bottles, "A Mother" advises the use of a weak solution of Condy, and we are sure many nurses who have the care of those very troublesome babies' bottles will be glad of the advice. Any help in keeping sweet the tubes and teats is always welcome. To keep litmus paper to test the acidity of the milk, and to add a proper quantity of lime-water, is another useful direction given by "A Mother." With regard to the use of Swiss milk, however, opinions differ, and perhaps "A Mother" recommends it a little too strongly. Although Swiss milk nourishes an infant, and many thrive upon it, it should never be entirely resorted to unless other milk is unobtainable. With regard to the dosing of infants, many authorities are quoted against it, and the experience of Dr. Semple that an experienced nurse can regulate a child's bowels by its food is vouched for. In cases of rickets the purely milk diet is forsaken earlier than usual, and meat juice and beef tea are given. The preparation of raw meat juice for a sick child is a duty which constantly falls to a nurse's share, and must be carefully done. Here Miss Wood's directions are the best, for she insists on the goodness and freshness of the meat used. Of course a nurse in a hospital has no chance of choosing the meat herself, and therefore, in private cases, is apt to leave the choice to others, a great mistake. Part of the undercut of a fresh sirloin of beef or quarter of a pound of the best rump steak should be used; cut it in slices and place in a small basin, with just sufficient cold water to cover it; a pinch of salt should be sprinkled over the meat. After soaking four hours squeeze the meat in muslin, press out all juice, which should be a bright red, and in quantity about two ounces; nothing but colourless fibre should be left in the muslin. This juice must always be prepared fresh every twelve hours, as it very quickly turns. It will be found to agree with children when all else fails. The recipes at the end of Miss Wood's book are all good, and we wish they were more numerous.

(*To be continued.*)

### Everybody's Opinion.

[*Correspondence on all subjects is invited, but we cannot in any way be responsible for the opinions expressed by our correspondents. No communications can be entertained if the name and address of the correspondent is not given, or unless one side of the paper only be written on.*]

### LADIES AS DISTRICT NURSES.

S. L. writes: I should like to say a few words to you on the subject of ladies as district nurses. I have worked for upwards of five years at an institution for district nursing where ladies only are received. I have never had the least trouble in getting on with the poor in their own homes—and in being able to sympathise with them. My visits have always been looked forward to with pleasure, and as far as my experience goes (and I have worked among all sorts of people, in town and country) I think a lady makes the best district nurse, and I am sure many will agree with me.

NURSE KATE writes: Ladies sometimes make good district nurses in towns, as your correspondents state, but certainly they don't make good district nurses in the country. Ask Miss Broadwood if she does not find "respectable women" work better and with less fuss? If the patients wrote to you instead of the lady nurses themselves we should hear a different story; it is easy to praise one's own work.

### A CO-OPERATIVE REGISTRY OFFICE FOR TRAINED NURSES.

ONE OF MANY writes: A few weeks ago you generously offered to help nurses to combine to receive their own earnings. The kind offer was not responded to, possibly because we are as yet too much in the power of the institutions. But the desire for justice is spreading, and could a small beginning be made, the nurses would join rapidly. Will you help us to start a registry office (supported by ourselves) in a central district? I have five friends, nurses, who make their home with me when disengaged, and we are connected with several others; our doctors send to us when they require nurses, and we get on very well, finding it both economical and pleasant to club together. Many more would combine with us were we supported by the Hospitals Association, or we are willing to co-operate in any plan proposed by those who have already done so much for us. We expect little from the B.N.A. There are too many matrons among them who appear to think that nurses should be mulcted for the benefit of the hospitals, though the public will scarcely be generous to the latter should it imagine they are gaining a large yearly sum by the labour of their nurses, who are thereby unable to save for that rainy day. The few medical men I have spoken to appear to be in favour of the movement, and I feel sure the profession, as a whole, would heartily support a registry office started and carried on by the nurses themselves. Probably the Hospitals Association would be able to find an educated and energetic nurse who would superintend the project, or should you, sir, be acquainted with any other band of united nurses, we shall be glad to amalgamate with them.—[We have ascertained that the hon. secs. of the Hospitals Association would be very glad to confer with "One of Many" and her friends, and those that agree with her proposal, if she will send us one or two alternative days and hours for a meeting to be held at the offices of the association, Norfolk House, opposite the Temple Station.]

### WHAT RISK WE SHOULD RUN.

A NURSE would like to hear the opinion of the majority on this question, How far should a nurse risk her life for the comfort of her patient in non-essentials? For example, a girl was dying at a hospital of a very fatal form of diphtheria. Two had already died of it, and the smell was perceptible outside the window. The only way the girl could take milk with any ease was when the nurse sat on the bed, supporting her with one arm, resting the patient's head on her shoulder. This the nurse did to the last, and contracted diphtheria of course, as she fully anticipated. This is only one of the many cases of the sort which constantly come in our way. There are so many little consolations for a dying or incurable case, which demand great personal risk from the nurse, such, for instance, as trying again and again to catch the faint attempt at a whisper, when the patient still thinks he is able to speak, and will try, and the nurse must either stand aloof and let her patient see that she is taking care of herself, or run a very grave risk with her face close to the mouth of her patient. The nurse who asks this question thinks that the least consolation for a patient is worth any risk to the nurse, coming in the way of duty as it does. Her friend, who is not a nurse, differs from her.

### Notices.

### HELP WANTED FOR A CRIPPLE.

CAN anyone tell me how to get £18 a year to keep a well-educated, superior woman (not a lady) out of the workhouse? She is terribly crippled by rheumatism, etc., and unable to earn her living.—Nurse V. Those willing to help should communicate with the Editor of THE HOSPITAL, The Lodge, Porchester-square, W.

### Appointments.

[It is requested that successful candidates will send a copy of their applications and testimonials, with date of election, to THE EDITOR, The Lodge, Porchester-square, W.]

HOSPITAL FOR EPILEPSY AND PARALYSIS.—The vacancy in the matronship at this hospital has been filled up by the appointment of Miss M. Ridley, who was selected by the committee from among several eligible candidates. Miss Ridley was trained at the Borough Hospital, Birkenhead, and at St. Bartholomew's, and has held the post of matron at the Cottage Hospital, Llandudno.

ALBANY HOSPITAL.—Miss Mary Magee, who trained at Leicester Infirmary, has been appointed matron of the Albany Hospital, Grahamstown, South Africa.

HOSPITAL FOR WOMEN, PATIALA.—The United Kingdom Branch of the National Association for Supplying Female Medical Aid to the Women of India has selected Miss Mary Crawley, L.R.C.P. and S. (Edinburgh), to the medical charge of this hospital, and as physician to the family of the Maharajah and those of his Sirdars. Miss Crawley was a distinguished student of the London School of Medicine for Women; she took her diploma in Edinburgh and has recently been employed as resident medical officer of the Hospital for Women and Children in Edinburgh.

### Notes and Queries.

TO CORRESPONDENTS.—1. Questions may be written on post-cards. 2. Advertisements in disguise are inadmissible. 3. In answering a query please quote the number. 4. If a private answer is desired, a stamped addressed envelope must be forwarded.

NOTICE.—We must really ask our correspondents to be more particular in enclosing name and address. Constantly we receive queries or letters with no name attached.

### Queries.

(18) *Disinfectants.*—What is the best disinfectant to use for a badly smelling sink? *Button.*

(19) *Annuity Fund.*—To whom should I apply for particulars of Lady Bloomfield's Trained Nurses' Annuity Fund? *M. A. P.*

(20) *Monthly Nursing.*—Is the month of the monthly nurse counted lunar or calendar? Where can pupil midwives be trained in the provinces, and for what fees? *H.*

### Answers.

(18) *Disinfectant.*—Sanitas Fluid, Jeyes', or Condy.

(19) *Annuity Fund.*—Apply to R. Gofton Salmond, Esq., hon. sec. 73, Cheapside, E.C.

*Probationer.*—We never insert anonymous letters. The cap seems to have fitted in your case.

*Leon.*—The verses are not quite correct enough in metre for publication.

*O. Smith and Miss E.*—The outward semblance may be that of a man, but internal evidence points to the work as emanating from one of the weaker sex.

*Irene.*—Yes, we increase our size with our circulation. You will notice that the other paper you mention has quietly reduced its size by some pages—a most significant fact.

*Frigid.*—Will "Frigid" please send her address? It has been mislaid, and several letters are waiting for her.

*Whitworth Hospital.*—We beg to inform "A. L. B." that so far as we can see there is no reference to the above hospital in our issue of Oct. 26.

*A Superintendent of District Nurses.*—Shortly; just now our columns are too full to open up the question.

### Presentations.

MISS HAMILTON, of Cove, Loch Long, and now senior sister at the Greek Hospital, Alexandria, has been decorated by the Khedive with the Khedive Bronze Star. Miss Hamilton trained at Westminster, and has worked at Alexandria since 1883.

THE probationer nurses of Bootle Borough Hospital have presented Dr. Atwood Beaver with a handsome clock, as a mark of esteem on the occasion of his resigning his office of house surgeon to the hospital. We are glad to say Dr. Beaver is the successful candidate out of 55 applicants for the post of house surgeon at Rainhill Asylum.

### Vacancies.

THE following vacancies are announced:

### Nurses.

Bath Royal United Hospital (four), £25.
Bedford Hospital Association.
Bedfordshire Institute, £25.
Belgrave Hospital for Children.
Birmingham Fever Hospital (several).
Blackheath Nursing Institute.
Bristol Infirmary (assistant).
Brixton Nursing Institute (two).
Carlisle Fever Hospital, £20.
City Chest Hospital, E., £20.
Clapham and Brixton Association.
Croydon General Hospital.
Firs Home, Bournemouth (night).
General Nursing Institute, Henrietta-street.
Greenock Infirmary.
Hampshire Nurses' Institute, £25.
Kidderminster Infirmary, £20.
King's Cross Hospital, Dundee (two), £24.
Leicester Fever Hospital.
Leicester Nurses' Institution, £25.
Liverpool City Hospital (assistant), £25.
Lynn Hospital, £20.
North Riding Infirmary, £20.
Norwich Nurses' Home.
Nursing Home, Bishops Stortford.
Nursing Institution, 96, 97, 98, Wimpole-street, London, W., Mr. Wilson has several vacancies for thoroughly trained nurses.
Paris Levick Institution.
Sheffield Nurses' Home (several).
South Devon Hospital.
St. George's Home, Sheffield.
St. Mark's Hospital, £18.
St. Pancras Workhouse, £20.
St. Saviour's Infirmary, £20.
Stockport Infirmary.
Sunderland Nursing Institute.
Swansea Nursing Institute.
The Wigmore Institution.
Truro Infirmary.
Tunbridge Wells Hospital, £25.
West Bromwich District Hospital, £25.
Workhouse Infirmary Nursing Association.

### District Nurses.

Bolton District Nurses' Home, £28.
Canterbury Nurses' Institute.
Hitchin Nursing Institution, £25.
Quorn Court, Loughborough, £65.
Stockport Infirmary, £25.

### Probationers.

Beckett Hospital, Barnsley.
Canterbury Hospital.
Maidstone Hospital.
Gateshead-on-Tyne Children's Hospital.
Truro Infirmary.

### Amusements and Relaxation.

**N.B. Third Quarterly Word Competition Commenced October 5th, 1889, ends December 28th, 1889.**

Three Prizes of 15s., 10s., 5s., will be given for the largest number of words derived from the words set for dissection.

Proper names, abbreviations, foreign words, words of less than four letters, and repetitions are barred; plurals, and past and present participles of verbs, are allowed. Nuttall's *Standard* dictionary only to be used.

N.B.—Word dissections must be sent in WEEKLY, arranged alphabetically, with correct total affixed.

The word for dissection for this, the *Sixth* week of the quarter, being "P I G E O N."

### Results of Fourth Week

| Names. | Oct. 31st. | Totals. | Names. | Oct. 31st. | Totals. |
|---|---|---|---|---|---|
| Winifred | 18 | 590 | Sunshine | — | 726 |
| Adelaide | 18 | 582 | Electrician | 23 | 846 |
| Nettie Vance | 22 | 663 | Reldas | 23 | 876 |
| F. W. P. H. | 23 | 864 | H. C. | — | 562 |
| Amos | 22 | 596 | Rover | — | 776 |
| Reyot | — | 369 | Beryl | — | 188 |
| A. S. K. | — | 275 | Qu'appelle | 21 | 720 |
| Ellice | 22 | 898 | Hercules | — | 12 |
| Neptune | 24 | 872 | Lightowlers | 22 | 934 |
| Ecila | 24 | 933 | Jenny Wren | 21 | 559 |
| Tinie | — | 17 | Oakenrod | 22 | 932 |
| Amicus | 20 | 870 | Gypsye | 20 | 781 |
| Juvenis | — | 539 | Leo | 17 | 610 |
| Sister | — | 438 | Night Watch | 15 | 346 |
| Jumps | 23 | 955 | Embryo | — | 385 |
| M. W. | 24 | 902 | Africando | 9 | 167 |
| Esperance | 24 | 906 | Pearl | 19 | 299 |

### Notice to Correspondents.

N.B.—All letters referring to this page which do not arrive at 139 and 140, Salisbury-court, London, E.C., by the *first post on Thursdays*, and are not addressed PRIZE EDITOR, will in future be disqualified and disregarded.

